# Association Between Immune‐Related Adverse Events and Treatment Outcomes in Advanced Gastric Cancer Patients Receiving Nivolumab Plus Chemotherapy: A Retrospective Study

**DOI:** 10.1002/cam4.71252

**Published:** 2025-09-17

**Authors:** Kazumasa Yamamoto, Hidekazu Hirano, Toshiharu Hirose, Hirokazu Shoji, Natsuko Okita, Atsuo Takashima, Ken Kato

**Affiliations:** ^1^ Department of Gastrointestinal Medical Oncology National Cancer Center Hospital Tokyo Japan; ^2^ Department of Medical Oncology Toranomon Hospital Tokyo Japan

**Keywords:** advanced gastric cancer, chemotherapy, immune checkpoint inhibitor (ICI), immune‐related adverse event (irAE)

## Abstract

**Background:**

Immune‐related adverse events (irAEs) have been linked to improved outcomes in patients undergoing treatment with immune checkpoint inhibitors (ICIs) for various cancers. However, the relationship between irAEs and overall survival (OS) in patients with advanced gastric cancer (AGC) receiving chemoimmunotherapy remains unclear. This study aimed to explore the association between irAEs and treatment outcomes in patients with AGC receiving chemotherapy plus nivolumab.

**Methods:**

This retrospective study analyzed clinical data from patients with HER2‐negative AGC who received first‐line chemotherapy (SOX, CapeOX, or FOLFOX) plus nivolumab at the National Cancer Center Hospital between November 2021 and February 2023. Patients were stratified into two groups based on the occurrence of irAEs. Outcomes, including OS and progression‐free survival (PFS), were compared using Kaplan–Meier analysis, landmark analysis, and Cox proportional hazards regression.

**Results:**

Among the 60 patients analyzed, 15 (25%) developed irAEs of any grade, with 3 patients (5%) experiencing grade ≥ 3 irAEs. Patients with irAEs had significantly longer OS and PFS in comparison to those without irAEs (median OS: not reached vs. 17.1 months, *p* < 0.01; median PFS: not reached vs. 6.8 months, *p* < 0.01). Multivariate analysis identified the occurrence of irAEs as a favorable prognostic factor for OS (hazard ratio: 0.13; 95% CI: 0.03–0.59; *p* < 0.01).

**Conclusion:**

This study suggests that the occurrence of irAEs is associated with improved survival outcomes in patients with AGC receiving chemotherapy plus nivolumab. IrAEs may serve as a predictive marker for treatment response in this setting.

## 1 Introduction

Gastric cancer ranks as the fifth most common cancer and is the fourth leading cause of cancer‐related death worldwide [1]. For patients with advanced gastric cancer (AGC), systemic treatment remains the primary approach, aiming to alleviate symptoms and extend survival. The advent of immune checkpoint inhibitors (ICIs), such as anti‐programmed death‐1 (PD‐1) antibodies, has been transformative in treating various types of cancer, including gastric cancer. In Japan, nivolumab was approved as a third‐ or later‐line treatment option for AGC in November 2017 based on the positive results of the ATTRACTION‐02 trial [2]. In the CheckMate‐649 trial, first‐line treatment with chemotherapy plus nivolumab demonstrated overall survival (OS) and progression‐free survival (PFS) benefits in comparison with chemotherapy alone in patients with AGC. These results led to the global approval of the first chemoimmunotherapy in the first‐line setting for AGC [3]. Subsequently, other pivotal clinical trials reproduced the survival benefits by adding anti‐PD‐1 antibodies (e.g., pembrolizumab, tislelizumab) to chemotherapy as the first‐line treatment for HER2‐negative AGC [4, 5].

The disruption of immunological tolerance exerted by ICIs is associated with immune‐related adverse events (irAEs), which may affect multiple organ systems [6]. Interestingly, previous studies have suggested a correlation between the development of irAEs and treatment efficacy in various cancers, including gastric cancer [7, 8, 9, 10, 11, 12, 13, 14, 15, 16, 17, 18]. In a previous study using landmark analysis, we reported that the development of irAEs was associated with longer survival in patients with AGC receiving nivolumab monotherapy in a third‐ or later‐line setting [14]. Although previous studies have reported an association between irAEs and survival outcomes in patients with gastric cancer treated with ICI monotherapy, especially in the third‐ or later‐line setting, data are scarce in the context of first‐line chemoimmunotherapy. Given the recent advancements in chemoimmunotherapy across various types of cancer, investigations into this topic are of significant importance.

Therefore, we retrospectively investigated the association between the development of irAEs and efficacy in patients with AGC receiving chemotherapy plus nivolumab in the first‐line setting.

## 2 Methods

### 2.1 Patients

We extracted the clinical data of patients with HER2‐negative AGC who underwent first‐line treatment with fluoropyrimidine plus oxaliplatin (S‐1 plus oxaliplatin [SOX], capecitabine plus oxaliplatin [CapeOX], or 5‐fluorouracil plus levofolinate plus oxaliplatin [FOLFOX]) combined with nivolumab at our institution between November 2021 and February 2023. All patients included in this study were Japanese and received treatment at the National Cancer Center Hospital in Tokyo, Japan. The selection criteria were as follows: age ≥ 18 years, Eastern Cooperative Oncology Group Performance Status (ECOG PS) 0–2, initially unresectable or recurrent disease, and histologically confirmed gastric or esophagogastric junctional carcinoma. Patients who had a history of prior treatment with ICIs were excluded. The study protocol was reviewed and approved by the institutional ethics committee of the National Cancer Center Hospital (approval number: 2017‐229). In accordance with our institution's opt‐out policy, patients were given the option to decline participation. Written informed consent was waived by the ethics committee because this was a retrospective study utilizing anonymized clinical data.

### 2.2 Treatment

Patients received nivolumab (240 mg every 2 weeks or 360 mg every 3 weeks) plus either FOLFOX (oxaliplatin 85 mg/m^2^, levofolinate 200 mg/m^2^, fluorouracil 400 mg/m^2^ bolus followed by 2400 mg/m^2^ over 46 h, every 2 weeks), SOX (oxaliplatin 130 mg/m^2^ on day 1 plus S‐1 40–60 mg twice daily on days 1–14, every 3 weeks), or CapeOX (oxaliplatin 130 mg/m^2^ on day 1 plus capecitabine 1000 mg/m^2^ twice daily on days 1–14, every 3 weeks) [3, 19]. Treatment was continued until disease progression, clinical deterioration, unacceptable toxicity, or patient refusal.

### 2.3 Assessment

The following patient characteristics were collected: age, sex, ECOG PS, histology, history of gastrectomy, metastatic sites, presence of measurable lesion according to Response Evaluation Criteria in Solid Tumors (RECIST) ver. 1.1, and baseline blood cell count and serum alkaline phosphatase (ALP) level before the initiation of first‐line chemotherapy. The neutrophil‐to‐lymphocyte ratio (NLR) was calculated by dividing the neutrophil count by the lymphocyte count.

Planned contrast‐enhanced or plain computed tomography was generally performed every 2–3 months. OS was calculated from the date of initiation of first‐line chemotherapy to the date of death or censored at the latest follow‐up for surviving patients. PFS was calculated from the date of initiation of first‐line chemotherapy to the date of disease progression or death, whichever came first, or censored at the latest follow‐up for surviving patients without disease progression. Tumor responses were assessed according to RECIST ver. 1.1. Only patients with measurable lesions were included in the ORR analysis. All patients, regardless of the presence of measurable lesions, were included in the analyses of PFS and OS.

Adverse events were evaluated according to the Common Terminology Criteria for Adverse Events (CTCAE) ver. 5.0. The irAEs evaluated in this study included cutaneous, gastrointestinal, endocrine, pulmonary, hepatobiliary, and renal AEs.

### 2.4 Statistical Analysis

We divided the patients into an irAE group and a non‐irAE group according to the presence of irAEs. Categorical variables were compared between the two groups using Fisher's exact test or the chi‐squared test. The chi‐squared test was used when expected frequencies were sufficient; otherwise, Fisher's exact test was applied. Continuous variables were compared with a *t*‐test or Mann–Whitney *U* test, depending on the distribution of the data. PFS and OS were estimated using the Kaplan–Meier method and compared between the groups using the log‐rank test. A landmark analysis was performed 4 months after the initiation of first‐line chemotherapy to address immortal time bias. The 4‐month time point was selected based on the observed median time to irAE onset, allowing for a balance between capturing sufficient irAE events and excluding early deaths or progressions. Univariate and multivariate analyses using a Cox proportional hazards regression model were performed to explore the impact of the occurrence of irAEs on PFS and OS. No imputation methods were applied for missing data. All statistical analyses were performed using EZR version 1.68 (Saitama Medical Center, Jichi Medical University, Saitama, Japan), which is a graphical user interface for R version 4.4.1 (The R Foundation for Statistical Computing, Vienna, Austria). EZR is a modified version of R commander designed to add statistical functions frequently used in biostatistics. The following R packages were used for the analyses: survival version 3.7‐0 (for Cox regression and survival analysis), ggplot2 version 3.5.1 (for data visualization), Rcmdr version 2.9‐2, and RcmdrPlugin. EZR version 1.68 (for GUI‐based statistical procedures). All *p* values are two‐sided, and *p* values of < 0.05 were considered to indicate statistical significance.

## 3 Results

### 3.1 Patient Characteristics and Profile of irAEs

The clinicopathological characteristics of 60 patients with AGC stratified by irAE development (irAE group, *n* = 15; non‐irAE group, *n* = 45) are shown in Table 1. There were no significant differences in the baseline characteristics of the irAE and non‐irAE groups. However, the irAE group exhibited a trend toward a higher eosinophil count in comparison to the non‐irAE group (median 150/μL vs. 100/μL, *p* = 0.051).

**TABLE 1:** Characteristics of patients in the irAE and non‐irAE groups.

	All patients	irAE	Non irAE	*p*
	*n* (%)	*n* (%)	*n* (%)	
Total *N*	60	15	45	
Age				
65 years	27 (45.0)	8 (53.3)	19 (42.2)	0.55
≥ 65 years	33 (55.0)	7 (46.7)	26 (57.8)	
Sex				
Female	21 (35.0)	3 (20.0)	18 (40.0)	0.22
Male	39 (65.0)	12 (80.0)	27 (60.0)	
ECOG PS				
0	20 (33.3)	7 (46.7)	13 (28.9)	0.22
≥ 1	40 (66.7)	8 (53.3)	32 (71.1)	
Disease status				
Stage IV	43 (71.7)	10 (66.7)	33 (73.3)	0.87
Recurrent	14 (23.3)	4 (26.7)	10 (22.2)	
Unresectable	3 (5.0)	1 (6.7)	2 (4.4)	
Peritoneal metastasis				
Absent	29 (48.3)	9 (60.0)	20 (44.4)	0.38
Present	31 (51.7)	6 (40.0)	25 (55.6)	
Volume of ascites				
None	35 (58.3)	10 (66.7)	25 (55.6)	0.39
Mild	12 (20.0)	4 (26.7)	8 (17.8)	
Moderate	5 (8.3)	0 (0)	5 (11.1)	
Massive	8 (13.3)	1 (6.7)	7 (15.6)	
Number of metastatic sites				
2	24 (40.0)	7 (46.7)	17 (37.8)	0.56
≥ 2	36 (60.0)	8 (53.3)	28 (62.2)	
Measurable lesion				
Present	38 (63.3)	8 (53.3)	30 (66.7)	0.37
Absent	22 (36.7)	7 (46.7)	15 (33.3)	
Histological type				
Intestinal	19 (31.7)	6 (40.00)	13 (28.9)	0.14
Diffuse	40 (66.7)	8 (53.3)	32 (71.1)	
Undifferentiated	1 (1.7)	1 (6.7)	0 (0)	
PD‐L1 CPS				
1	19 (31.7)	6 (40.0)	13 (28.9)	0.74
1–5	13 (21.7)	4 (26.7)	9 (20.0)	
≥ 5	22 (36.7)	4 (26.7)	18 (40.0)	
NE	6 (10.0)	1 (6.7)	5 (11.1)	
MSI status				
MSS	23 (38.3)	7 (46.7)	16 (35.6)	0.54
NE	37 (61.7)	8 (53.3)	29 (64.4)	
ALP				
Low (< 113 U/L)	45 (75.0)	13 (86.7)	32 (71.1)	0.31
High (≥ 113 U/L)	15 (25.0)	2 (13.3)	13 (28.9)	
NLR				
Low (< 4)	39 (65.0)	11 (73.3)	28 (62.2)	0.54
High (≥ 4)	21 (35.0)	4 (26.7)	17 (37.8)	
Baseline blood cell count median (range)				
WBC (/μL)	5850 (2400–14,500)	5900 (3600–13,300)	5800 (2400–14,500)	0.27
Neutrophil (/μL)	4205 (1170–11,240)	3880 (2490–11,240)	4260 (1170–10,740)	0.38
Lymphocyte (/μL)	1270 (470–2800)	1430 (620–2690)	1210 (470–2800)	0.30
Eosinophil (/μL)	110 (0–550)	150 (0–550)	100 (0–330)	0.051
NLR	3.30 (0.66–9.37)	3.05 (1.84–9.37)	3.38 (0.67–9.36)	0.49

Abbreviations: ALP, alkaline phosphatase; CPS, combined positive score; ECOG PS, Eastern Cooperative Oncology Group performance status; irAE, immune‐related adverse event; MSI, microsatellite instability; NLR, neutrophil/lymphocyte ratio; WBC, white blood cell.

### 3.2 Profile of irAEs

Table 2 presents the profile of irAEs. The frequency of irAEs was 33.3% (1/3) in the CapeOX plus nivolumab group, 28.6% (12/42) in the SOX group, and 13.3% (2/15) in the FOLFOX group. The most common irAEs were adrenal insufficiency (*n* = 7, 46.7%), skin rash (*n* = 3, 20.0%), colitis (*n* = 2, 13.3%), and pneumonitis (*n* = 2, 13.3%). Most irAEs were grade 1 or 2 (*n* = 12, 80.0%). Grade 3–4 irAEs occurred in 3 patients (20.0%). The median time from the initiation of first‐line chemotherapy to the onset of irAEs was 8.0 (range: 2.8–19.8) months. The median time to onset varied by irAE type: 3.7 months (2.8–7.5) for skin rash, 7.1 months for type 1 diabetes mellitus, 7.9 months (5.4–10.4) for colitis, 9.8 months (8.0–11.6) for pneumonitis, and 14.3 months (6.6–19.8) for adrenal insufficiency. For patients with colitis and pneumonitis, systemic steroid therapy with prednisolone (1 mg/kg) was administered as initial treatment. For skin rash, a topical steroid ointment was used, and for adrenal insufficiency, hydrocortisone replacement therapy was provided. Insulin therapy was initiated for type 1 diabetes mellitus.

**TABLE 2:** Profiles of irAEs in the irAE group.

irAE	Any grade	Grade 1–2	Grade 3–4	Median time to onset
	*n* (%)	*n* (%)	*n* (%)	(months, range)
Total	15 (100)	12 (80.0)	3 (20.0)	8.0 (2.8–19.8)
Adrenal insufficiency	7 (46.7)	7 (46.7)	0	14.3 (6.6–19.8)
Skin rash	3 (20.0)	3 (20.0)	0	3.7 (2.8–7.5)
Colitis	2 (13.3)	1 (6.7)	1 (6.7)	7.9 (5.4–10.4)
Pneumonitis	2 (13.3)	1 (6.7)	1 (6.7)	9.8 (8.0–11.6)
Type 1 diabetes mellitus	1 (6.7)	0	1 (6.7)	7.1 (7.1–7.1)

Abbreviation: irAE, immune‐related adverse event.

### 3.3 Comparison of Survival Outcomes Between the irAE and Non‐irAE Groups

The median follow‐up was 18.6 months (interquartile range, 14.1–21.2). Overall, 39 patients (65.0%) had disease progression during first‐line chemotherapy plus nivolumab, and 23 patients (38.3%) died during follow‐up.

**FIGURE 1:**
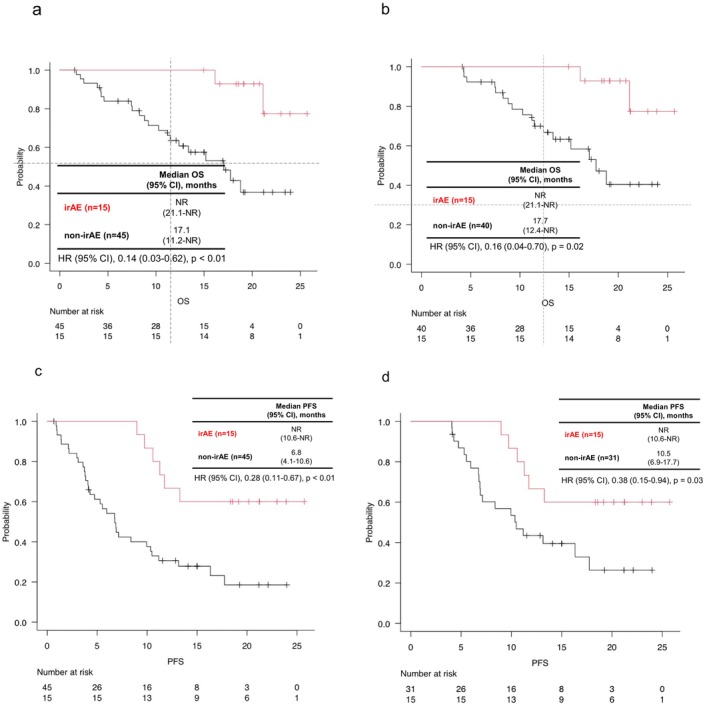
Overall survival (OS) and progression‐free survival (PFS) in patients with and without irAEs. (a) OS in the irAE (red line) and non‐irAE (black line) groups. (b) Landmark analysis of OS at 4 months. (c) PFS in the irAE and non‐irAE groups. (d) Landmark analysis of PFS at 4 months. HR (95% CI), 0.14 (0.03–0.62), *p* < 0.01.

The median OS in the irAE group was significantly longer than that in the non‐irAE group (median OS: [21.1–not reached (NR)] vs. 17.1 [11.2–NR] months, hazard ratio = 0.14 (95% CI [confidence interval]: 0.03–0.62), *p* < 0.01; Figure 1a). Additionally, we conducted a landmark analysis at 4 months, excluding patients who experienced events within 4 months (Figure 1b). This analysis revealed that OS was significantly longer in the irAE group (median OS: NR [21.1–NR] vs. 17.7 [12.4–NR] months, hazard ratio = 0.16 [95% CI: 0.04–0.70], *p* = 0.02). To further assess the robustness of our findings, we conducted additional landmark analyses at 2, 6, and 8 months (Figure S1). Across all landmark time points, OS was consistently longer in the irAE group compared to the non‐irAE group. In the 2‐month landmark analysis, the median OS was not reached in the irAE group (95% CI: 21.1–NR), whereas it was 17.1 months (95% CI: 11.5–NR) in the non‐irAE group (HR = 0.15 [95% CI: 0.03–0.64]; *p* = 0.01). In the 4‐month analysis, the median OS was not reached in the irAE group (95% CI, 21.1–NR) and was 18.8 months (95% CI, 13.4–NR) in the non‐irAE group (HR = 0.18 [95% CI, 0.04–0.80]; *p* = 0.02). The 6‐month landmark analysis yielded consistent findings (NR [95% CI, 21.1–NR] vs. 18.8 months [95% CI, 15.2–NR]; HR, 0.20 [95% CI: 0.04–0.89]; *p* = 0.03).

The median PFS was significantly longer in the irAE group than in the non‐irAE group (median PFS: NR [10.6–NR] vs. 6.8 [4.1–10.6] months, hazard ratio = 0.28 [95% CI: 0.11–0.67], *p* < 0.01; Figure 1c). The landmark analysis at 4 months showed that PFS was significantly longer in the irAE group (median PFS: NR [10.6–NR] vs. 10.5 [6.9–17.7] months, hazard ratio = 0.38 (95% CI: 0.15–0.94), *p* = 0.03; Figure 1d). Next, we performed additional landmark analyses at 2, 6, and 8 months (Figure S2). Although the superiority in PFS in the irAE group tended to attenuate at later landmark time points, a favorable trend was consistently observed across all analyses compared with the non‐irAE group. At the 2‐month landmark analysis, the median PFS was not reached in the irAE group (95% CI: 10.6–NR) and was 7.1 months (95% CI: 5.3–11.2) in the non‐irAE group (HR = 0.30 [95% CI: 0.12–0.74]; *p* < 0.01). At the 4‐month landmark, the median PFS was not reached (95% CI: 10.6–NR) versus 16.3 months [95% CI: 10.0–NR] (HR = 0.52 [95% CI: 0.19–1.37]; *p* = 0.18). At the 8‐month landmark, the median PFS was not reached (95% CI: 10.6–NR) versus 17.7 months (95% CI: 11.2–NR) (HR = 0.78 [95% CI: 0.27–2.27]; *p* = 0.65).

Table 3 shows the best overall response according to the development of irAEs. Among patients with measurable lesions, the objective response rates (ORRs) in patients with and without irAEs were 37.5% and 62.1%, respectively (*p* = 0.25). None of the patients in the irAE group had a best overall response of PD.

**TABLE 3:** Best overall response to first‐line chemotherapy plus nivolumab in patients with measurable lesions.

	All patients	irAE	non‐irAE	
	*n* (%)	*n* (%)	*n* (%)	
CR	1 (2.7)	1 (12.5)	0	
PR	20 (54.1)	2 (25.0)	18 (62.1)	
SD	11 (29.7)	5 (62.5)	6 (20.7)	
PD	5 (13.5)	0	5 (17.2)	
				***p***
ORR (%)	56.8	37.5	62.1	0.25
DCR (%)	86.5	100	82.8	0.56

Abbreviations: CR, complete response; DCR, disease control rate; irAE, immune‐related adverse event; ORR, overall response rate; PD, progressive disease; PR, partial response; SD, stable disease.

### 3.4 Impact of Lesion Measurability on Survival Outcomes

To explore the potential impact of lesion measurability on survival outcomes, we compared both OS and PFS between patients with measurable and non‐measurable lesions (Figure S3a,b). The median OS was 21.1 months (95% CI, 15.2–NR) in patients without measurable lesions (*n* = 22) and not reached (95% CI, 12.4–NR) in those with measurable lesions (*n* = 38), with no significant difference (HR = 1.31 [95% CI: 0.55–3.01], *p* = 0.54). The median PFS was 13.3 months (95% CI, 5.5–NR) and 7.1 months (95% CI, 4.7–11.7) in patients with and without measurable lesions, respectively (HR = 1.59 [95% CI: 0.80–3.15], *p* = 0.18).

### 3.5 Univariate and Multivariate Analyses of Survival Outcomes

In the univariate analysis, the development of irAEs was significantly associated with longer OS (hazard ratio = 0.14 (95% CI: 0.03–0.62), *p* < 0.01). In the multivariate analysis, the development of irAEs was identified as a favorable prognostic factor for OS (hazard ratio = 0.13 [95% CI: 0.03–0.59], *p* < 0.01) (Table 4).

**TABLE 4:** Univariate and multivariate analyses for OS.

Variable	Category	Event/*N*	Univariate analysis		Multivariate analysis	
			HR (95% CI)	*p*	HR (95% CI)	*p*
irAE	Absent	21/45	ref.	< 0.01	ref.	< 0.01
	Present	2/15	0.14 (0.03–0.62)		0.13 (0.03–0.59)	
ECOG PS	0	4/20	ref.	0.03	ref.	0.62
	≥ 1	19/40	3.37 (1.14–9.99)		1.37 (0.40–4.73)	
Number of metastatic sites	< 2	6/24	ref.	0.04	ref.	0.53
	≥ 2	17/36	2.65 (1.04–6.74)		1.46 (0.45–4.73)	
Serum ALP	Non‐elevated (≤ 113 U/L)	14/45	ref.	0.01	ref.	0.15
	Elevated (> 113 U/L)	9/15	3.02 (1.29–7.07)		2.28 (0.74–7.03)	
Disease status	Recurrence	2/14	ref.		ref.	
	Stage IV	20/43	4.49 (1.04–19.27)	0.04	4.22 (0.84–21.11)	0.08
	Unresectable	1/3	2.83 (0.25–31.40)	0.40	7.47 (0.51–108.8)	0.14
NLR	Low (< 4)	11/39	ref.	< 0.01	ref.	0.20
	High (≥ 4)	12/21	3.12 (1.37–7.10)		1.98 (0.70–5.64)	

Abbreviations: ALP, alkaline phosphatase; ECOG PS, Eastern Cooperative Oncology Group performance status; irAE, immune‐related adverse event; NLR, neutrophil/lymphocyte ratio.

In the univariate analysis, the development of irAEs was significantly associated with longer PFS (hazard ratio = 0.28 [95% CI: 0.11–0.67], *p* < 0.01). In the multivariate analysis, the development of irAEs remained an independent favorable prognostic factor for PFS (hazard ratio = 0.25 [95% CI: 0.10–0.63], *p* < 0.01) (Table 5).

**TABLE 5:** Univariate and multivariate analyses for PFS.

Variable	Category	Event/*N*	Univariate analysis		Multivariate analysis	
			HR (95% CI)	*p*	HR (95% CI)	*p*
irAE	Absent	33/45	ref.	< 0.01	ref.	< 0.01
	Present	6/15	0.28 (0.11–0.67)		0.25 (0.10–0.63)	
ECOG PS	0	10/20	ref.	0.10	ref.	0.45
	≥ 1	29/40	1.82 (0.89–3.75)		1.38 (0.60–3.21)	
Number of metastatic sites	< 2	11/24	ref.	< 0.01	ref.	0.01
	≥ 2	28/36	3.04 (1.50–6.16)		3.07 (1.30–7.27)	
Serum ALP	Non‐elevated (≤ 113 U/L)	26/45	ref.	< 0.01	ref.	0.33
	Elevated (> 113 U/L)	13/15	2.73 (1.39–5.38)		1.48 (0.67–3.27)	
Disease status	Recurrence	9/14	ref.		ref.	
	Stage IV	29/43	1.08 (0.51–2.28)	0.84	0.75 (0.31–1.84)	0.53
	Unresectable	1/3	0.36 (0.05–2.84)	0.33	0.60 (0.07–5.40)	0.65
NLR	Low (< 4)	22/39	ref.	0.02	ref.	0.23
	High (≥ 4)	17/21	2.20 (1.16–4.17)		1.55 (0.75–3.20)	

Abbreviations: ALP, alkaline phosphatase; ECOG PS, Eastern Cooperative Oncology Group performance status; irAE, immune‐related adverse event; NLR, neutrophil/lymphocyte ratio.

To assess the robustness of the association between NLR and survival outcomes, we performed sensitivity analyses using different NLR cut‐off values (3 and 5) (Tables S1–S4). In univariate analysis, a higher NLR (≥ 3 or ≥ 5) was associated with worse OS (HR: 2.77 [95% CI: 1.03–7.47] and 3.18 [1.30–7.77], respectively) and PFS (HR: 1.98 [0.98–3.98] and 1.92 [0.91–4.06], respectively). In multivariate models adjusting for irAE occurrence, ECOG PS, and other clinical covariates, these associations weakened (OS: HR = 1.14 [0.33–4.02] and 2.14 [0.71–6.44]; PFS: HR = 0.72 [0.28–1.82] and 1.31 [0.58–2.96], respectively), but the overall trends remained consistent.

### 3.6 Number of Treatment Cycles for Each Drug

The median number of treatment cycles was analyzed for patients receiving CapeOX + nivolumab, SOX + nivolumab, and FOLFOX + nivolumab. For the CapeOX + nivolumab or SOX + nivolumab regimens, the median number of cycles for S‐1 or capecitabine in the irAE group (15.0 cycles) was significantly higher than that in the non‐irAE group (6.5 cycles) (*p* < 0.01). A similar trend was observed for nivolumab (15.0 cycles vs. 6.0 cycles, *p* < 0.01) and oxaliplatin (8.0 cycles vs. 6.0 cycles, *p* = 0.02). For the FOLFOX + nivolumab regimen, the irAE group showed a higher median number of cycles for 5‐FU (20.5 cycles vs. 8.0 cycles, *p* = 0.15), nivolumab (20.5 cycles vs. 8.0 cycles, *p* = 0.15), and oxaliplatin (11.5 cycles vs. 8.0 cycles, *p* = 0.44) in comparison to the non‐irAE group (Table 6).

**TABLE 6:** Number of treatment cycles for each drug.

CapeOX+Nivo or SOX+Nivo, median cycle (range)				
	All	irAE group	Non‐irAE group	*p*
	(*n* = 45)	(*n* = 13)	(*n* = 32)	
S‐1 or capecitabine	10 (1–33)	15 (10–33)	6.5 (1–21)	< 0.01
Oxaliplatin	7 (1–17)	8 (5–17)	6 (1–14)	0.02
Nivolumab	9 (1–33)	15 (10–33)	6 (1–21)	< 0.01

FOLFOX+Nivo, median cycle (range)				
	All	irAE group	Non‐irAE group	*p*
	(*n* = 15)	(*n* = 2)	(*n* = 13)	
5FU	10 (2–38)	20.5 (19–22)	8 (2–38)	0.15
Oxaliplatin	8 (2–38)	11.5 (8–15)	8 (2–38)	0.44
Nivolumab	8 (2–34)	20.5 (19–22)	8 (2–34)	0.15

Abbreviation: irAE, immune‐related adverse event.

### 3.7 Non‐irAE Toxicities of First‐Line Chemotherapy

Common treatment‐related adverse events of any grade included peripheral neuropathy (63.3%), anemia (95.0%), thrombocytopenia (65.0%), neutropenia (63.3%), increased aspartate aminotransferase (AST) (63.3%), and increased alanine aminotransferase (ALT) (50.0%). No significant differences in incidences of grade ≥ 3 adverse events were observed between the irAE and non‐irAE groups (Table 7).

**TABLE 7:** Non‐irAE treatment‐related toxicities.

	Any grade				Grade ≥ 3			
	All	irAE group	Non‐irAE group	*p*	All	irAE group	Non‐irAE group	*p*
	(*n* = 60)	(*n* = 15)	(*n* = 45)		(*n* = 60)	(*n* = 15)	(*n* = 45)	
Peripheral neuropathy	38 (63.3%)	15 (100%)	23 (51.1%)	< 0.01	1 (1.7%)	1 (6.7%)	0	0.25
Diarrhea	16 (26.7%)	5 (33.3%)	11 (24.4%)	0.52	0	0	0	NA
Hand‐foot syndrome	8 (13.3%)	5 (33.3%)	3 (6.7%)	0.02	0	0	0	NA
Mucositis	6 (10.0%)	2 (13.3%)	4 (8.9%)	0.63	0	0	0	NA
Nausea	17 (28.3%)	4 (26.7%)	13 (28.9%)	1.00	0	0	0	NA
Vomiting	5 (8.3%)	0	5 (11.1%)	0.32	0	0	0	NA
Anorexia	27 (45.0%)	7 (46.7%)	20 (44.4%)	1.00	1 (1.7%)	0	1 (2.2%)	1.00
Fatigue	30 (50.0%)	4 (26.7%)	26 (57.8%)	0.07	0	0	0	NA
Constipation	34 (56.7%)	7 (46.7%)	27 (60.0%)	0.39	0	0	0	NA
Leukopenia	29 (48.3%)	5 (33.3%)	24 (53.3%)	0.24	8 (13.3%)	0	8 (17.8%)	0.18
Anemia	57 (95.0%)	15 (100%)	42 (93.3%)	0.57	5 (8.3%)	0	5 (11.1%)	0.32
Thrombocytopenia	39 (65.0%)	13 (86.7%)	26 (57.8%)	0.06	2 (3.3%)	1 (6.7%)	1 (2.2%)	0.44
Neutropenia	38 (63.3%)	12 (80.0%)	26 (57.8%)	0.22	17 (28.3%)	1 (6.7%)	16 (35.6%)	0.05
Febrile neutropenia	3 (5.0%)	0	3 (6.7%)	0.57	3 (5.0%)	0	3 (6.7%)	0.57
Increased AST	38 (63.3%)	12 (80.0%)	26 (57.8%)	0.22	3 (5.0%)	0	3 (6.7%)	0.57
Increased ALT	30 (50.0%)	8 (53.3%)	22 (48.9%)	1.00	4 (6.7%)	0	4 (8.8%)	0.56
Increased bilirubin	15 (25.0%)	4 (26.7%)	11 (24.4%)	1.00	0	0	0	NA
Renal dysfunction	15 (25.0%)	4 (26.7%)	11 (24.4%)	1.00	0	0	0	NA

Abbreviations: ALT, alanine aminotransferase; AST, aspartate aminotransferase; NA, not applicable.

## 4 Discussion

To the best of our knowledge, this is the first study to explore the relationship between irAEs and OS in patients with AGC receiving chemoimmunotherapy. Previous studies on irAEs in AGC have primarily focused on ICI monotherapy in later‐line settings; however, our study extends these findings to the context of first‐line chemoimmunotherapy. Our study demonstrated that patients who developed irAEs had better survival outcomes than those without irAEs. The association between the development of irAEs and longer OS remained significant even after conducting a landmark analysis. To support the validity of our 4‐month landmark analysis, we conducted additional landmark analyses at 2, 6, and 8 months. The results consistently showed longer overall survival in patients with irAEs across all time points, supporting the robustness of our findings. In contrast, the favorable association between irAE development and PFS tended to attenuate at later landmark time points, which may be partly sustained by treatment response in the non‐irAE group.

The underlying mechanism of irAEs is thought to be the destruction of autologous cells and tissues by autoantibodies and the inadvertent activation of autoantigen‐specific lymphocytes that are produced after administration of ICIs and remain in the body without being removed [20]. It has been suggested that ICIs recognize an antigen that is shared between tumors and hosts, which leads to the occurrence of irAEs [21]. A case study on myocarditis and myositis showed expanded T‐cell receptor (TCR)‐β sequences in paired heart, tumor, and muscle tissue, suggesting the presence of a common antigen shared between the irAE tissue and tumor [22]. Subsequent studies further characterized the role of autoreactive T cells recognizing tumor‐associated antigens in the context of skin rash [23, 24, 25] and pneumonitis [26].

There is increasing evidence to suggest that the occurrence of irAEs is associated with better treatment outcomes with ICIs. However, a similar association regarding chemoimmunotherapy has not yet been sufficiently investigated. In non‐small cell lung cancer, there are a few reports supporting the association between the development of irAEs and favorable prognosis in patients treated with chemoimmunotherapy [16, 17]. In this study, we demonstrated that in patients with AGC receiving chemoimmunotherapy, the occurrence of irAEs is also associated with favorable clinical outcomes. Specifically, patients who developed irAEs had significantly longer OS (median, NR vs. 17.1 months, *p* < 0.01) and PFS (median, NR vs. 6.8 months, *p* < 0.01) compared to those without irAEs. Multivariate analysis further identified the occurrence of irAEs as an independent favorable prognostic factor. Our findings suggest that the occurrence of irAEs could serve as a universal prognostic marker of favorable outcomes across different treatment modalities, including ICI monotherapy and chemoimmunotherapy. This underscores the importance of monitoring irAEs in clinical practice as a useful indicator for evaluating treatment efficacy. In this study, although the difference was not statistically significant, the ORR was lower in the irAE group than in the non‐irAE group. This may be partly attributed not only to the small sample size. Notably, none of the patients in the irAE group had a best overall response of PD in this study. These findings suggest that patients who develop irAEs and achieve a best overall response of SD or better may sustain an effective immune response that contributes to antitumor activity.

Although these findings are based on a small number of cases and should be interpreted with caution, they suggest that specific irAE phenotypes or underlying immune contexts may contribute to deeper tumor regression. These results provide preliminary insights that may inform future research directions.

Furthermore, while no significant differences in baseline characteristics were observed between the irAE and non‐irAE groups, the irAE group completed a significantly greater number of treatment cycles. The increased number of completed treatment cycles in the irAE group suggests better treatment tolerance and sustained drug exposure, which may have directly contributed to the improved treatment outcomes. Nevertheless, it is important to note that the higher number of treatment cycles in the irAE group may be partially attributable to longer survival time and delayed disease progression, rather than a direct causal effect of irAE occurrence. That said, the significantly longer PFS observed in the irAE group indicates that the benefits extend beyond merely maintaining the duration of treatment. This suggests that the occurrence of irAEs reflects an enhanced immune response that contributes to tumor control independently of the duration of treatment. These findings underscore the critical importance of effective irAE management to ensure treatment continuity and maximize therapeutic efficacy. Oxaliplatin has been shown to induce immunogenic cell death and modulate systemic immune responses by enhancing CD8^+^ T cell activation and increasing TNF‐α expression [27]. In addition, combination therapy with oxaliplatin and ICIs has been associated with an increased incidence of immune‐mediated and dermatologic adverse events, such as rash and hypersensitivity reactions [28]. Indirect comparisons across different clinical trials also suggest that the frequency of adverse events appears higher with chemoimmunotherapy than with nivolumab monotherapy. However, it should be noted that in the chemoimmunotherapy setting, distinguishing irAEs from chemotherapy‐related toxicities may be inherently challenging.

In our study, we observed that 25% of patients experienced any‐grade irAEs, while 5% experienced grade ≥ 3 irAEs, aligning closely with the reported ranges of 24%–31% for any‐grade irAEs and 5%–18% for grade ≥ 3 irAEs reported in previous clinical trials, including CheckMate‐649 [3], RATIONALE‐305 [5], KEYNOTE‐859 [4], ATTRACTION‐4 [19], and KEYNOTE‐062 [29]. The median time to the onset of irAEs in this study was 8.0 months, which was longer than previously reported. The median time to the onset of adverse events due to PD‐1/PD‐L1 inhibitors has been reported as follows: skin, 8.4 weeks (95% CI: 7.7–9.1); gastrointestinal, 6.1 weeks (95% CI: 5.7–9.1); hepatic, 8.9 weeks (95% CI: 7.7–11.1); endocrine, 12.3 weeks (95% CI: 8.0–16.0); and pulmonary, 12.0 weeks (95% CI: 8.2–12.1) [30]. The longer onset times for any‐grade irAEs in our study may reflect the naturally wide range of onset periods characteristic of irAEs. However, this delay may also be influenced by multiple factors, including variability in clinical recognition and documentation, differences in patient backgrounds or comorbidities, and inconsistencies in the definitions of irAE onset across studies. Several studies have shown that an increase in baseline eosinophil levels is a risk factor for the development of irAEs, and in our study, a similar trend was observed, although the difference was not statistically significant [31, 32, 33, 34, 35].

This study had several limitations. Firstly, this study was a retrospective, single‐center study involving a small number of Japanese patients. Although this study showed the association of irAE development and treatment outcomes in Japanese patients treated with first‐line chemoimmunotherapy, comparable data from non‐Japanese cohorts are not yet available. The generalizability of our findings to other ethnic populations remains uncertain. Nonetheless, the incidence and severity of irAEs, as well as the response to ICIs, may be influenced by ethnic differences [36, 37]. Further investigations in diverse populations are needed to confirm these results and to identify potential interethnic differences. While there are reports indicating the prognostic impact of the severity and type of irAEs, the small sample size of our study makes it challenging to analyze irAEs by severity or type. Due to the inherent limitations of a single‐center, retrospective study, there is a possibility of selection bias and variability in clinical practice. Future prospective, multicenter studies with standardized data collection protocols are warranted to validate and expand upon our results.

In conclusion, our study demonstrated that the development of irAEs was associated with favorable survival outcomes in patients with AGC receiving chemoimmunotherapy in the first‐line setting.

## Author Contributions

**Kazumasa Yamamoto:** conceptualization, investigation, formal analysis, writing – original draft, writing – review and editing. **Hidekazu Hirano:** conceptualization, methodology, investigation, formal analysis, writing – review and editing. **Toshiharu Hirose:** writing – review and editing. **Hirokazu Shoji:** writing – review and editing. **Natsuko Okita:** writing – review and editing. **Atsuo Takashima:** writing – review and editing. **Ken Kato:** writing – review and editing, supervision.

## Ethics Statement

The study protocol was reviewed and approved by the Institutional Ethics Committee of the National Cancer Center Hospital (approval number: 2017‐229). In accordance with the institution's opt‐out policy, patients were informed about the study and given the opportunity to decline the research use of their clinical data. Written informed consent was waived by the Institutional Ethics Committee due to the retrospective nature of the study and the use of an opt‐out approach.

## Conflicts of Interest

K.Y. and N.O. have no conflicts of interest. H.H. has received honoraria from Novartis, Ono Pharmaceutical, Taiho Pharmaceutical, and Bristol‐Myers Squibb and grants from PPD, Daiichi‐Sankyo, Nippon Boehringer Ingelheim, ALX Oncology, BeiGene, Novartis, Amgen, Bristol‐Myers Squibb, and Taiho Pharmaceutical to the institution outside the submitted work. T.H. has received honoraria from Lilly, Taiho Pharmaceutical, Bristol‐Myers Squibb, and Ono Pharmaceutical and grants from Lilly, to the institution outside the submitted work. H.S. has received honoraria from Ono Pharmaceutical, Zymeworks, Astellas Pharma, and Amgen and grants from Ono Pharmaceutical, Takeda Pharmaceutical, MSD, Astellas Pharma, Amgen, Daiichi‐Sankyo, AbbVie, AstraZeneca, Taiho Pharmaceutical, and Elevation Oncology, to the institution outside the submitted work. A.T. has received honoraria from Lilly, Ono Pharmaceutical, Taiho Pharmaceutical, Chugai Pharma, Takeda, and Merck Serono and grants from Incyte Biosciences Japan, Eisai, Ono Pharmaceutical, Seagen Inc. and Pfizer Japan, to the institution outside the submitted work. K.K. reports funding to the institution from Merck Sharp & Dohme Corp (MSD), Ono Pharmaceuticals, Bristol Myers Squibb (BMS), Beigene, Shionogi, Merck Biopharma, Oncolys BioPharma, Daiichi Sankyo, Novartis, Taiho Pharmaceutical, Janssen, AstraZeneca, and Chugai.

## Supporting information


**Appendix S1:** Supporting Information.

## Data Availability

The data that support the findings of this study is available from the corresponding author upon reasonable request.
