# Supplementary material for: Association Between Immune‐Related Adverse Events and Treatment Outcomes in Advanced Gastric Cancer Patients Receiving Nivolumab Plus Chemotherapy: A Retrospective Study
Source: Cancer Med. 2025 Sep 17;14(18):e71252. doi: 10.1002/cam4.71252 (PMC12441737; doi:10.1002/cam4.71252)
Supplement: Supplementary file 1 — Appendix S1: Supporting Information. [file CAM4-14-e71252-s001.docx]

Supplemental material for

**Association Between Immune-Related Adverse Events and Treatment Outcomes in Advanced Gastric Cancer Patients Receiving Nivolumab Plus Chemotherapy: A Retrospective Study**

Kazumasa Yamamoto, Hidekazu Hirano, Toshiharu Hirose, Hirokazu Shoji, Natsuko Okita, Atsuo Takashima and Ken Kato

**Contents**

Supplemental table 1

Supplemental table 2

Supplemental table 3

Supplemental table 4

Supplemental figure 1

Supplemental figure 2

Supplemental figure 3

**Supplemental table 1.**

Univariate and multivariate analyses for OS using NLR cut-off of 3

| **Variable** | **Category** | **Event/N** | **Univariate analysis** | | **Multivariate analysis** | |
| --- | --- | --- | --- | --- | --- | --- |
|  |  |  | **HR (95% CI)** | **P-value** | **HR (95% CI)** | **P-value** |
| irAE | Absent | 21/45 | ref. | <0.01 | ref. | <0.01 |
|  | Present | 2/15 | 0.14 (0.03-0.62) |  | 0.12 (0.03-0.57) |  |
| ECOG PS | 0 | 4/20 | ref. | 0.03 | ref. | 0.43 |
|  | ≥ 1 | 19/40 | 3.37 (1.14-9.99) |  | 1.66 (0.48-5.78) |  |
| Number of metastatic sites | < 2 | 6/24 | ref. | 0.04 | ref. | 0.46 |
|  | ≥ 2 | 17/36 | 2.65 (1.04-6.74) |  | 1.55 (0.49-4.92) |  |
| Serum ALP | Non-elevated (≤ 113 U/L) | 14/45 | ref. | 0.01 | ref. | 0.054 |
|  | Elevated (> 113 U/L) | 9/15 | 3.02 (1.29-7.07) |  | 2.89 (0.98-8.51) |  |
| Disease status | Recurrence | 2/14 | ref. |  | ref. |  |
|  | Stage Ⅳ | 20/43 | 4.49 (1.04-19.27) | 0.04 | 4.19 (0.88-19.91) | 0.07 |
|  | Unresectable | 1/3 | 2.83 (0.25-31.40) | 0.40 | 6.37 (0.44-92.87) | 0.18 |
| NLR | Low (< 3) | 5/22 | ref. | 0.04 | ref. | 0.83 |
|  | High (≥ 3) | 18/38 | 2.77 (1.03-7.47) |  | 1.14 (0.33-4.02) |  |

irAE, immune-related adverse event; ECOG PS, Eastern Cooperative Oncology Group performance status; ALP, alkaline phosphatase; NLR, neutrophil/lymphocyte ratio

**Supplemental table 2.**

Univariate and multivariate analyses for PFS using NLR cut-off of 3

| **Variable** | **Category** | **Event/N** | **Univariate analysis** | | **Multivariate analysis** | |
| --- | --- | --- | --- | --- | --- | --- |
|  |  |  | **HR (95% CI)** | **P-value** | **HR (95% CI)** | **P-value** |
| irAE | Absent | 33/45 | ref. | <0.01 | ref. | <0.01 |
|  | Present | 6/15 | 0.28 (0.11-0.67) |  | 0.24 (0.10-0.61) |  |
| ECOG PS | 0 | 10/20 | ref. | 0.10 | ref. | 0.19 |
|  | ≥ 1 | 29/40 | 1.82 (0.89-3.75) |  | 1.83 (0.75-4.44) |  |
| Number of metastatic sites | < 2 | 11/24 | ref. | <0.01 | ref. | <0.01 |
|  | ≥ 2 | 28/36 | 3.04 (1.50-6.16) |  | 3.38 (1.38-8.28) |  |
| Serum ALP | Non-elevated (≤ 113 U/L) | 26/45 | ref. | <0.01 | ref. | 0.16 |
|  | Elevated (> 113 U/L) | 13/15 | 2.73 (1.39-5.38) |  | 1.78 (0.80-3.98) |  |
| Disease status | Recurrence | 9/14 | ref. |  | ref. |  |
|  | Stage Ⅳ | 29/43 | 1.08 (0.51-2.28) | 0.84 | 0.73 (0.30-1.77) | 0.49 |
|  | Unresectable | 1/3 | 0.36 (0.05-2.84) | 0.33 | 0.43 (0.05-4.00) | 0.46 |
| NLR | Low (< 3) | 11/22 | ref. | 0.06 | ref. | 0.48 |
|  | High (≥ 3) | 28/38 | 1.98 (0.98-3.98) |  | 0.72 (0.28-1.82) |  |

irAE, immune-related adverse event; ECOG PS, Eastern Cooperative Oncology Group performance status; ALP, alkaline phosphatase; NLR, neutrophil/lymphocyte ratio

**Supplemental table 3.**

Univariate and multivariate analyses for OS using NLR cut-off of 5

| **Variable** | **Category** | **Event/N** | **Univariate analysis** | | **Multivariate analysis** | |
| --- | --- | --- | --- | --- | --- | --- |
|  |  |  | **HR (95% CI)** | **P-value** | **HR (95% CI)** | **P-value** |
| irAE | Absent | 21/45 | ref. | <0.01 | ref. | <0.01 |
|  | Present | 2/15 | 0.14 (0.03-0.62) |  | 0.12 (0.03-0.55) |  |
| ECOG PS | 0 | 4/20 | ref. | 0.03 | ref. | 0.43 |
|  | ≥ 1 | 19/40 | 3.37 (1.14-9.99) |  | 1.62 (0.49-5.31) |  |
| Number of metastatic sites | < 2 | 6/24 | ref. | 0.04 | ref. | 0.45 |
|  | ≥ 2 | 17/36 | 2.65 (1.04-6.74) |  | 1.56 (0.49-4.93) |  |
| Serum ALP | Non-elevated (≤ 113 U/L) | 14/45 | ref. | 0.01 | ref. | 0.18 |
|  | Elevated (> 113 U/L) | 9/15 | 3.02 (1.29-7.07) |  | 2.22 (0.70-7.07) |  |
| Disease status | Recurrence | 2/14 | ref. |  | ref. |  |
|  | Stage Ⅳ | 20/43 | 4.49 (1.04-19.27) | 0.04 | 3.97 (0.80-19.65) | 0.09 |
|  | Unresectable | 1/3 | 2.83 (0.25-31.40) | 0.40 | 6.31 (0.44-89.70) | 0.17 |
| NLR | Low (< 5) | 16/48 | ref. | 0.01 | ref. | 0.18 |
|  | High (≥ 5) | 7/12 | 3.18 (1.30-7.77) |  | 2.14 (0.71-6.44) |  |

irAE, immune-related adverse event; ECOG PS, Eastern Cooperative Oncology Group performance status; ALP, alkaline phosphatase; NLR, neutrophil/lymphocyte ratio

**Supplemental table 4.**

Univariate and multivariate analyses for PFS using NLR cut-off of 5

| **Variable** | **Category** | **Event/N** | **Univariate analysis** | | **Multivariate analysis** | |
| --- | --- | --- | --- | --- | --- | --- |
|  |  |  | **HR (95% CI)** | **P-value** | **HR (95% CI)** | **P-value** |
| irAE | Absent | 33/45 | ref. | <0.01 | ref. | <0.01 |
|  | Present | 6/15 | 0.28 (0.11-0.67) |  | 0.25 (0.10-0.62) |  |
| ECOG PS | 0 | 10/20 | ref. | 0.10 | ref. | 0.30 |
|  | ≥ 1 | 29/40 | 1.82 (0.89-3.75) |  | 1.54 (0.69-3.48) |  |
| Number of metastatic sites | < 2 | 11/24 | ref. | <0.01 | ref. | 0.01 |
|  | ≥ 2 | 28/36 | 3.04 (1.50-6.16) |  | 3.02 (1.30-7.02) |  |
| Serum ALP | Non-elevated (≤ 113 U/L) | 26/45 | ref. | <0.01 | ref. | 0.29 |
|  | Elevated (> 113 U/L) | 13/15 | 2.73 (1.39-5.38) |  | 1.54 (0.69-3.43) |  |
| Disease status | Recurrence | 9/14 | ref. |  | ref. |  |
|  | Stage Ⅳ | 29/43 | 1.08 (0.51-2.28) | 0.84 | 0.74 (0.30-1.82) | 0.51 |
|  | Unresectable | 1/3 | 0.36 (0.05-2.84) | 0.33 | 0.52 (0.06-4.59) | 0.56 |
| NLR | Low (< 5) | 30/48 | ref. | 0.09 | ref. | 0.52 |
|  | High (≥ 5) | 9/12 | 1.92 (0.91-4.06) |  | 1.31 (0.58-2.96) |  |

irAE, immune-related adverse event; ECOG PS, Eastern Cooperative Oncology Group performance status; ALP, alkaline phosphatase; NLR, neutrophil/lymphocyte ratio

**Supplemental figure 1.** Overall Survival (OS) in Patients With and Without irAEs

(a) Landmark analysis of OS at 2 months. (b) Landmark analysis of OS at 6 months. (c) Landmark analysis of OS at 8 months.

**
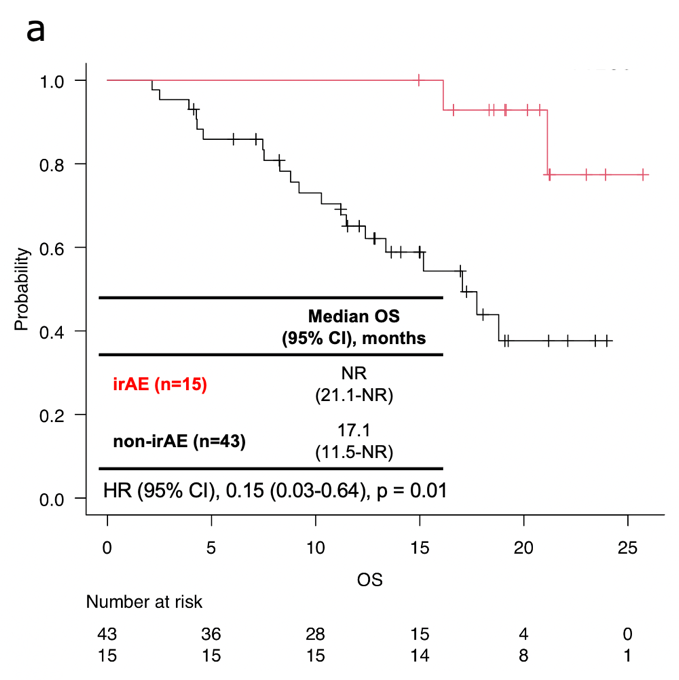

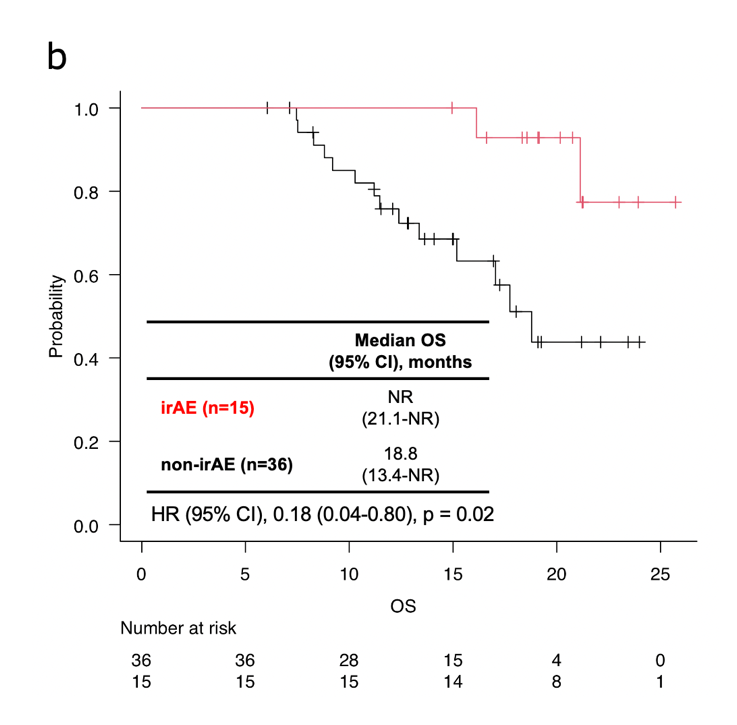

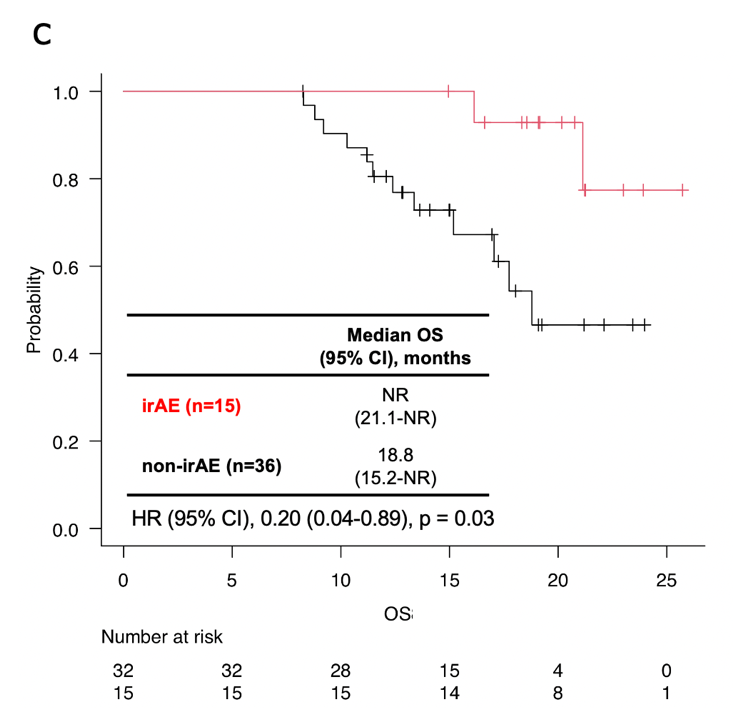
**

CI, confidence interval; HR, hazard ratio; n, number; OS, overall survival.

**Supplemental figure 2.** Progression-Free Survival (PFS) in Patients With and Without irAEs

(a) Landmark analysis of PFS at 2 months. (b) Landmark analysis of PFS at 6 months. (c) Landmark analysis of PFS at 8 months.


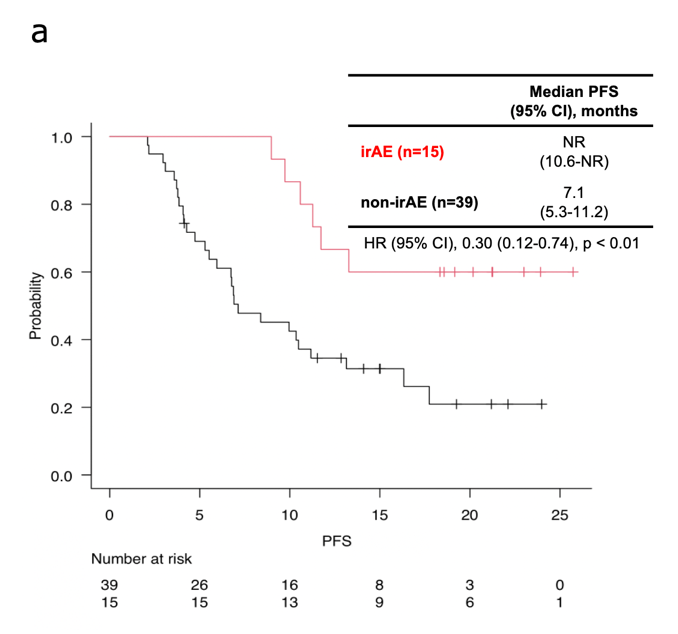

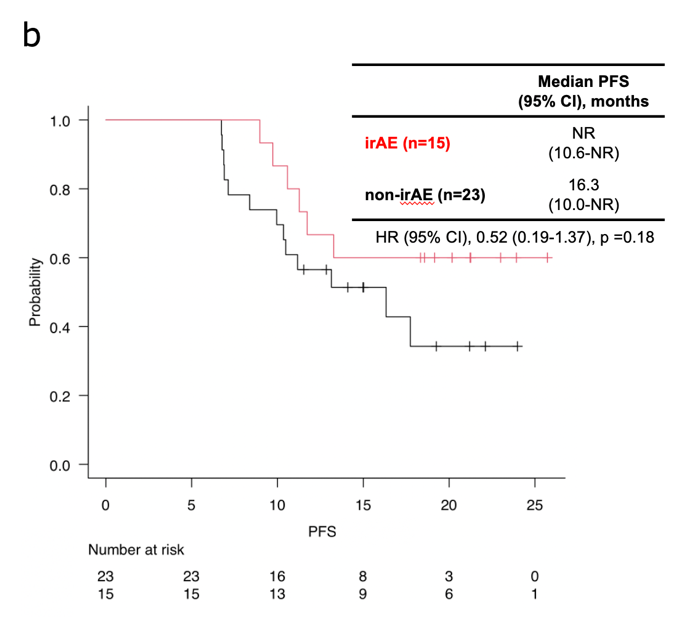

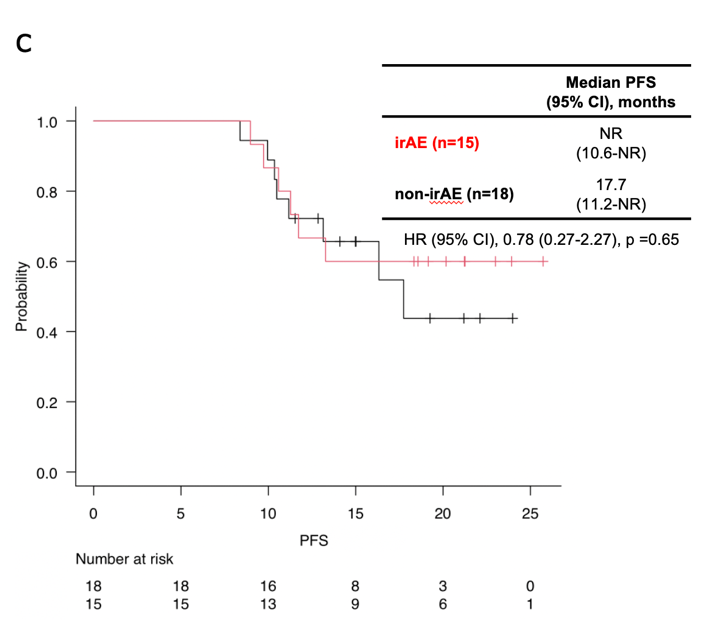


CI, confidence interval; HR, hazard ratio; n, number; PFS, progression-free survival.

**Supplemental figure 3.** Overall Survival (OS) and Progression-Free Survival (PFS) in Patients With and Without measurable lesions

(a) OS in the patients with (red line) and without (black line) measurable lesions

(b) PFS in the patients with and without measurable lesions


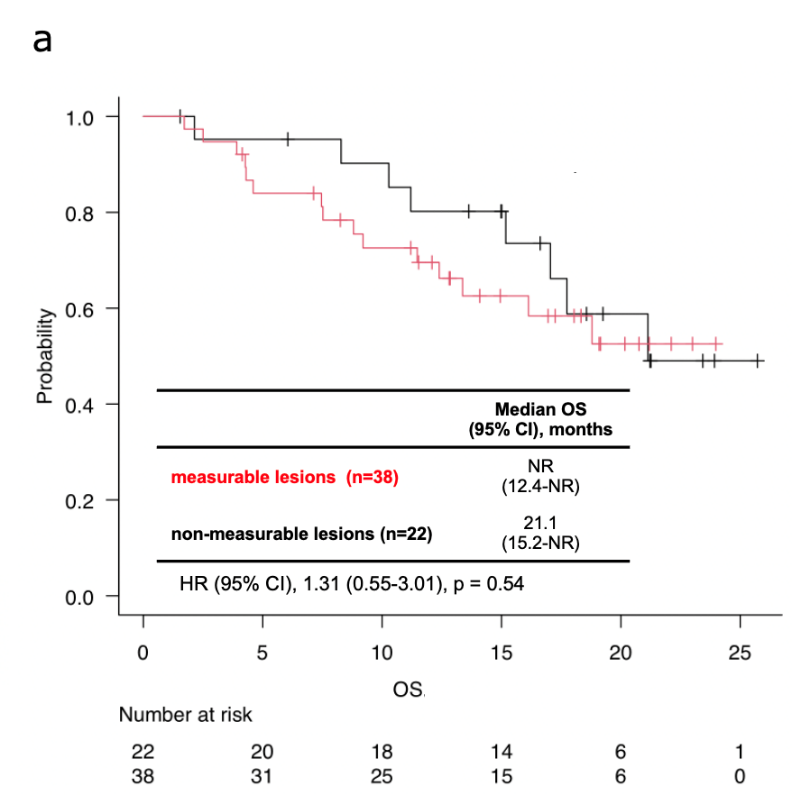

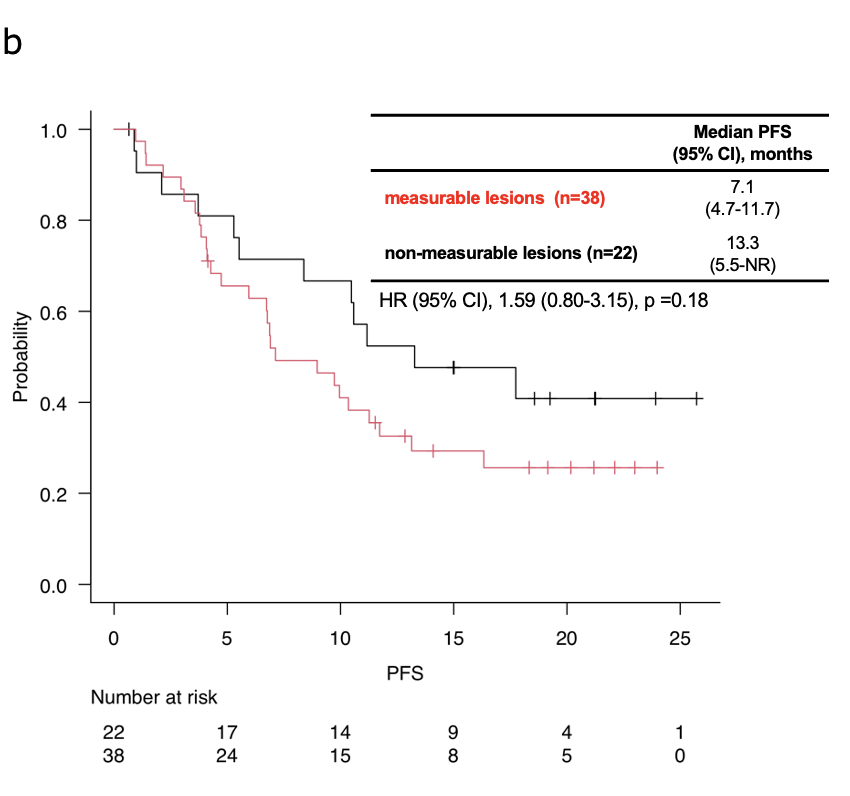


CI, confidence interval; HR, hazard ratio; n, number; PFS, progression-free survival; OS, overall survival.
